# Shedding light on the base-pair opening dynamics of nucleic acids in living human cells

**DOI:** 10.1038/s41467-022-34822-4

**Published:** 2022-11-29

**Authors:** Yudai Yamaoki, Takashi Nagata, Keiko Kondo, Tomoki Sakamoto, Shohei Takami, Masato Katahira

**Affiliations:** 1 Institute of Advanced Energy, Kyoto University, Uji, Kyoto 611-0011 Japan; 2 Graduate School of Energy Science, Kyoto University, Uji, Kyoto 611-0011 Japan; 3 Integrated Research Center for Carbon Negative Science, Institute of Advanced Energy, Kyoto University, Uji, 611-0011 Japan; 4 Biomass Product Tree Industry-Academia Collaborative Research Laboratory, Kyoto University, Uji, Kyoto 611-0011 Japan

**Keywords:** Solution-state NMR, Nucleic acids

## Abstract

Base-pair opening is a fundamental property of nucleic acids that plays important roles in biological functions. However, studying the base-pair opening dynamics inside living cells has remained challenging. Here, to determine the base-pair opening kinetics inside living human cells, the exchange rate constant (${k}_{{{{{{\rm{ex}}}}}}}$) of the imino proton with the proton of solvent water involved in hairpin and G-quadruplex (GQ) structures is determined by the in-cell NMR technique. It is deduced on determination of ${k}_{{{{{{\rm{ex}}}}}}}$ values that at least some G-C base pairs of the hairpin structure and all G-G base-pairs of the GQ structure open more frequently in living human cells than in vitro. It is suggested that interactions with endogenous proteins could be responsible for the increase in frequency of base-pair opening. Our studies demonstrate a difference in dynamics of nucleic acids between in-cell and in vitro conditions.

## Abstract (web summary)

Base-pair opening is important for nucleic acids to exert biological functions, but studying its dynamics inside living cells is challenging. Here, the authors determine the base-pair opening kinetics of hairpin and G-quadruplex structures inside living human cells by the in-cell NMR technique, and demonstrate a difference in dynamics of nucleic acids between in-cell and in vitro conditions.

## Introduction

The intracellular environment is very crowded with macromolecules, which causes a reduction of water activity, and increases in the excluded volume effect, viscosity, and specific/non-specific interactions^1–4^. Therefore, the structure, dynamics, and interactions of nucleic acids are considered to be different under in vitro and in-cell conditions. To determine the actual properties of nucleic acids under physiologically relevant conditions, analysis of nucleic acids inside the living cell is essential.

A base pair is a fundamental unit of the nucleic acid structure. The opening of the base pair is reportedly related to various biological events through modulation of the interactions between nucleic acids and proteins^5–8^. Nevertheless, the fundamental properties, such as the frequency of base-pair opening, in living cells remain unclear. To analyze the behavior of base pairs of nucleic acids in living cells at atomic resolution, the in-cell NMR technique is a potential tool^4,9–12^. To date, by using the in-cell NMR technique, the formation of DNA and RNA hairpins^13^, and i-motif^14,15^, DNA GQ^16^, DNA:RNA hybrid GQ^17^, Z-DNA^18^, and DNA triplex^19^ structures was revealed in living human cells. Additionally, the interactions of nucleic acids with small compounds^20–22^, and those of mRNA with modified nucleic acids^23^ have also been studied in living human cells. However, observation of the intracellular dynamics of a base pair has not previously been achieved.

**Fig. 1:**
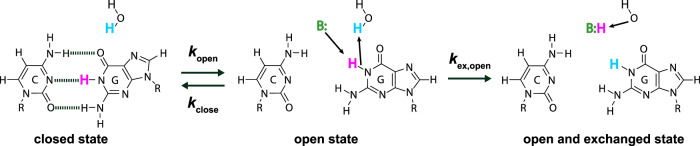
Imino proton exchange reaction. There are “closed”, “open”, and “open and exchanged” states. The rate constants for base-pair opening, ${k}_{{{{{{\rm{open}}}}}}}$, base-pair closing, ${k}_{{{{{{\rm{close}}}}}}}$, and imino proton exchange at the open state catalyzed by a base catalyst (B:), ${k}_{{{{{{\rm{ex}}}}}},{{{{{\rm{open}}}}}}}$, are indicated. Hydrogen bonds are shown by dashed lines.

Here, we incorporate water magnetization transfer measurement^8,24,25^ to in-cell NMR experiments to analyze the rate of exchange of the imino proton with the proton of solvent water in living human cells. The imino proton exchange rate gives information on the opening dynamics of individual base-pairs in living human cells. Exchange of the imino proton with the proton of solvent water does not occur in the closed state (Fig. 1, left), but it does in the open state by means of a base catalyst (Fig. 1, center and right). Therefore, the overall imino proton exchange rate, ${k}_{{{{{{\rm{ex}}}}}}}$, is expressed using the rate constants for base-pair opening, ${k}_{{{{{{\rm{open}}}}}}}$, base-pair closing, ${k}_{{{{{{\rm{close}}}}}}}$, and imino proton exchange in the open state catalyzed by a base catalyst, ${k}_{{{{{{\rm{ex}}}}}},{{{{{\rm{open}}}}}}}$^25^.

1 $${k}_{{{{{{\rm{ex}}}}}}}=\frac{{k}_{{{{{{\rm{open}}}}}}}\,\cdot \,{k}_{{{{{{\rm{ex}}}}}},{{{{{\rm{open}}}}}}}}{{k}_{{{{{{\rm{close}}}}}}}+{k}_{{{{{{\rm{ex}}}}}},{{{{{\rm{open}}}}}}}}$$

In this study, we determine this overall ${k}_{{{{{{\rm{ex}}}}}}}$ for each base pair in nucleic acids exogenously introduced into living human cells (${k}_{{{{{{\rm{ex}}}}}}}^{{{{{{\rm{in}}}}}}-{{{{{\rm{cell}}}}}}}$), after which the range of ${k}_{{{{{{\rm{open}}}}}}}$ in the living human cells (${k}_{{{{{{\rm{open}}}}}}}^{{{{{{\rm{in}}}}}}-{{{{{\rm{cell}}}}}}}$) is deduced. The ${k}_{{{{{{\rm{open}}}}}}}$ under in-cell conditions turns out to be larger than that under in vitro conditions for many residues. It is indicated that at least some G-C base-pairs of the hairpin structure and all G-G base-pairs of the GQ structure open more frequently in living human cells than in vitro. Thus, the base-pair opening dynamics is different between in vitro and in living human cells. Analysis with various crowding agents under in vitro conditions suggests that the increase in the frequency of base-pair opening in living human cells is due to the interaction of nucleic acids with positively charged proteins inside the cells.

## Results

### The imino proton exchange rate of the hairpin structure in living human cells

**Fig. 2:**
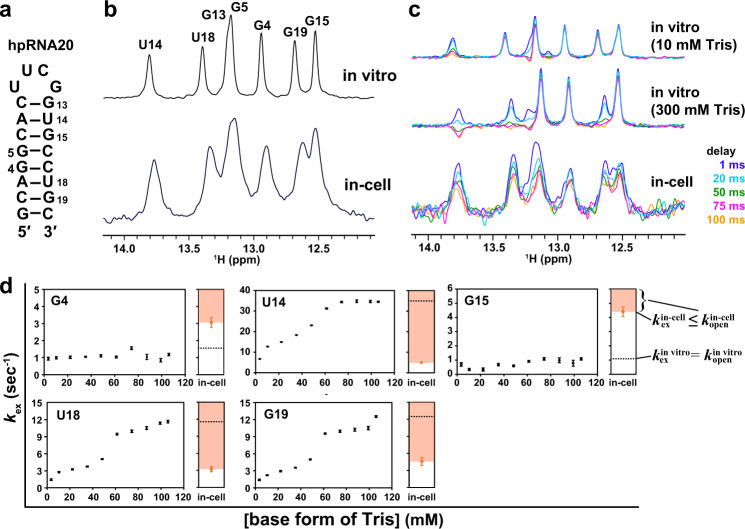
Analysis of the exchange of imino protons of the RNA hairpin structure with the proton of solvent water. **a** Secondary structure of fully 2′-*O*-methylated hpRNA20. **b** 1D ^1^H-NMR spectra of hpRNA20 recorded under in vitro (upper) and in-cell (lower) conditions. **c** Water magnetization transfer experiments on hpRNA20 carried out under two in vitro conditions, 10 mM (top) and 300 mM Tris (middle), and under in-cell conditions for living HeLa cells (bottom). Spectra recorded with different delay times after water-selective inversion are superimposed. **d** The comparison of the deduced ${k}_{{{{{{\rm{open}}}}}}}$ values of hpRNA20 between in-cell and in vitro conditions by using the ${k}_{{{{{{\rm{ex}}}}}}}$ value as a reference. For each residue, the dashed line indicates the maximum ${k}_{{{{{{\rm{ex}}}}}}}$ value for various concentrations of the base form of Tris. The maximum ${k}_{{{{{{\rm{ex}}}}}}}$ value is regarded to be equal to ${k}_{{{{{{\rm{open}}}}}}}^{{{{\rm{in}}}}\; {{{\rm{vitro}}}}}$ (see text for details). The orange shading indicates the deduced ${k}_{{{{{{\rm{open}}}}}}}^{{{{{{\rm{in}}}}}}-{{{{{\rm{cell}}}}}}}$ value range for each residue of hpRNA20 in HeLa cells (see text for details). For each base form of Tris concentration, the intensity of imino proton was obtained for each residue (*n* = 1). Standard deviation of the noise signal for the region of 1D ^1^H spectrum in which no signal is present was calculated and used as error bars of intensities. The means and error bars of the ${k}_{{{{{{\rm{ex}}}}}}}$ values were obtained from 50 data sets of intensities constructed by Monte Carlo simulation using the error bars of intensities. Data are presented as mean values ± standard deviation. Source data are provided as a Source Data file.

We used a hairpin RNA (hpRNA20: 5′-GCAGGCACUUCGGUGCCUGC-3′, fully 2′-*O*-methylated, Fig. 2a) containing a stable UUCG tetra-loop, which was originally found in ribosomal RNAs and the P1 helix of group I intron^26–28^. The imino proton regions of 1D NMR (Fig. 2b, upper) and 2D NOESY spectra (Supplementary Fig. 1) of hpRNA20 measured under in vitro conditions indicated that hpRNA20 forms a hairpin structure, as shown in Fig. 2a.

Firstly, the base-pair opening kinetics of hpRNA20 under in vitro conditions was evaluated by imino proton exchange rate analysis. 1D NMR spectra obtained in water magnetization transfer experiments measured with different delay times are superimposed in Fig. 2c (top and middle). Briefly, in this experiment, a selective 180° pulse for the proton of solvent water was applied and after various delay times (ranging from 1 to 100 ms), the imino proton signal was acquired. The signal intensity of each imino proton was plotted as a function of the delay time and ${k}_{{{{{{\rm{ex}}}}}}}^{{{{{{\rm{in\; vitro}}}}}}}$ was calculated by curve fitting (Eq. (3), “Methods” section)^25^. To determine the ${k}_{{{{{{\rm{open}}}}}}}^{{{{{{\rm{in\; vitro}}}}}}}$, as explained below, the ${k}_{{{{{{\rm{ex}}}}}}}^{{{{{{\rm{in\; vitro}}}}}}}$ values were obtained for various concentrations of tris(hydroxymethyl)aminomethane (Tris) (Fig. 2d, Table 1). Here, we define the “concentration of the base form of Tris” as the concentration of (HOCH_2_)_3_CNH_2_. We also define the “total concentration of Tris” as the sum of the concentrations of (HOCH_2_)_3_CNH_2_ and (HOCH_2_)_3_CNH_3_^+^. The base form of Tris acts as a base catalyst that accelerates the imino proton exchange in the open state, thus increasing ${k}_{{{{{{\rm{ex}}}}}},{{{{{\rm{open}}}}}}}$ (Fig. 1). When ${k}_{{{{{{\rm{ex}}}}}},{{{{{\rm{open}}}}}}}\gg {k}_{{{{{{\rm{close}}}}}}}$ is satisfied at a high concentration of the base form of Tris, the denominator in Eq. (1) can be replaced by ${k}_{{{{{{\rm{ex}}}}}},{{{{{\rm{open}}}}}}}$, and thereby ${k}_{{{{{{\rm{ex}}}}}}}$ is equal to ${k}_{{{{{{\rm{open}}}}}}}$, ${k}_{{{{{{\rm{ex}}}}}}}$ being independent of the concentration of the base form of Tris.

**Table 1:** ${k}_{{{{{{\rm{ex}}}}}}}^{{{{{{\rm{in\; vitro}}}}}}}$ and ${k}_{{{{{{\rm{ex}}}}}}}^{{{{{{\rm{in}}}}}}-{{{{{\rm{cell}}}}}}}$ of hpRNA20

	${k}_{{{{{{\rm{ex}}}}}}}^{{{{{{\rm{in\; vitro}}}}}}}$ [s^−1^] (10 mM Tris)	${k}_{{{{{{\rm{ex}}}}}}}^{{{{{{\rm{in\; vitro}}}}}}}$ [s^−1^] (300 mM Tris)	${k}_{{{{{{\rm{ex}}}}}}}^{{{{{{\rm{in}}}}}}-{{{{{\rm{cell}}}}}}}$ [s^−1^]
**G4**	0.9 ± 0.1	1.2 ± 0.1	3.0 ± 0.8
**U14**	6.7 ± 0.1	34.5 ± 0.3	6.2 ± 0.4
**G15**	0.7 ± 0.1	1.1 ± 0.1	3.2 ± 0.8
**U18**	1.4 ± 0.1	12.5 ± 0.2	4.2 ± 0.7
**G19**	1.4 ± 0.1	11.6 ± 0.2	6.7 ± 0.7

Figure 2d indicates that at around 100 mM base form of Tris, the ${k}_{{{{{{\rm{ex}}}}}}}$ of each nucleobase (G or U) reaches the maximum and is almost independent of the concentration of the base form of Tris. 100 mM base form of Tris corresponds to ca. 300 mM total concentration of Tris (see Methods for reference). Therefore, it is supposed that ${k}_{{{{{{\rm{ex}}}}}}}$ at 300 mM of the total concentration of Tris equals ${k}_{{{{{{\rm{open}}}}}}}$. Therefore, the second column in Table 1 indicates that U14, U18 and G19 exhibit large ${k}_{{{{{{\rm{open}}}}}}}^{{{{{{\rm{in\; vitro}}}}}}}$ values, the largest being that of U14. On the other hand, the second column in Table 1 also indicates that G4 and G15 have small ${k}_{{{{{{\rm{open}}}}}}}^{{{{{{\rm{in\; vitro}}}}}}}$. Generally, a G-C base-pair is more stable and harder to open than an A-U base-pair. Additionally, base-pairs located away from the end of the stem region open less frequently than ones located near the end, because the former are less affected by the end-fraying effect. The G-C base-pairs involving G4 and G15 are located near the center of the stem region. Therefore, it is reasonable that the base-pairs involving G4 and G15 exhibit much lower ${k}_{{{{{{\rm{open}}}}}}}^{{{{{{\rm{in\; vitro}}}}}}}$ values than those involving U14, U18, and G19. As described in the Methods section, the pH was adjusted to within the range of 7.9 and 8.2. It should be noted that although the Tris concentration is increased, the pH remained the same as described in detail in the Methods section.

Next, hpRNA20 was introduced into living HeLa cells using a pore-forming toxin, SLO, after which the pores on a cell membrane were resealed using CaCl_2_^13,29–31^. Comparison of the imino proton regions of 1D NMR spectra of hpRNA20 acquired under in vitro and in-cell conditions indicated that hpRNA20 forms the same hairpin structure under both conditions (Fig. 2b, upper and lower).

We then carried out a water magnetization transfer experiment on the imino protons of hpRNA20 introduced into living HeLa cells, analyzed the imino proton exchange rate, and determined the base-pair opening kinetics. The signal intensities of the imino protons decreased with increasing delay time (Fig. 2c, bottom). The signal intensities were plotted as a function of the delay time (Supplementary Fig. 2), and the imino proton exchange rate, ${k}_{{{{{{\rm{ex}}}}}}}^{{{{{{\rm{in}}}}}}-{{{{{\rm{cell}}}}}}}$, was determined (Table 1, Fig. 2d). The order of the magnitude of the ${k}_{{{{{{\rm{ex}}}}}}}^{{{{{{\rm{in}}}}}}-{{{{{\rm{cell}}}}}}}$ values was G19$\approx$U14$>$U18$>$G15$\approx$G4.

As the next step, we compared ${k}_{{{{{{\rm{open}}}}}}}$, the rate constant for base-pair opening, between in vitro and in-cell conditions. It should be noted that ${k}_{{{{{{\rm{ex}}}}}}}^{{{{{{\rm{in}}}}}}-{{{{{\rm{cell}}}}}}}$ and ${k}_{{{{{{\rm{ex}}}}}}}^{{{{{{\rm{in\; vitro}}}}}}}$ cannot be compared because the total concentration of base catalysts in the living human cell is not known. Here, we evaluated the range of ${k}_{{{{{{\rm{open}}}}}}}^{{{{{{\rm{in}}}}}}-{{{{{\rm{cell}}}}}}}$ by using the ${k}_{{{{{{\rm{ex}}}}}}}^{{{{{{\rm{in}}}}}}-{{{{{\rm{cell}}}}}}}$ value as a reference. The underlying principle is as follows. Transformation of Eq. (1) results in the following equation.

$$\frac{{k}_{{{{{{\rm{ex}}}}}}}}{{k}_{{{{{{\rm{open}}}}}}}}=\frac{{k}_{{{{{{\rm{ex}}}}}},{{{{{\rm{open}}}}}}}}{{k}_{{{{{{\rm{close}}}}}}}+{k}_{{{{{{\rm{ex}}}}}},{{{{{\rm{open}}}}}}}}\le 1$$

$$\frac{{k}_{{{{{{\rm{ex}}}}}}}}{{k}_{{{{{{\rm{open}}}}}}}}\le 1$$

2 $${k}_{{{{{{\rm{ex}}}}}}}\le {k}_{{{{{{\rm{open}}}}}}}$$

This equation indicates that ${k}_{{{{{{\rm{open}}}}}}}^{{{{{{\rm{in}}}}}}-{{{{{\rm{cell}}}}}}}$ is always larger than or equal to ${k}_{{{{{{\rm{ex}}}}}}}^{{{{{{\rm{in}}}}}}-{{{{{\rm{cell}}}}}}}$. Therefore, ${k}_{{{{{{\rm{open}}}}}}}^{{{{{{\rm{in}}}}}}-{{{{{\rm{cell}}}}}}}$ of a base pair takes a value within the range depicted as the orange shading of panels in Fig. 2d.

In the case of G4 and G15, ${k}_{{{{{{\rm{ex}}}}}}}^{{{{{{\rm{in}}}}}}-{{{{{\rm{cell}}}}}}}$ (Fig. 2d, orange dot) was much larger than ${k}_{{{{{{\rm{open}}}}}}}^{{{{{{\rm{in\; vitro}}}}}}}$ (Fig. 2d, black dashed line), which was obtained as the maximum ${k}_{{{{{{\rm{ex}}}}}}}^{{{{{{\rm{in\; vitro}}}}}}}$ when the concentration of the base form of Tris was varied. This indicates that the ${k}_{{{{{{\rm{open}}}}}}}^{{{{{{\rm{in}}}}}}-{{{{{\rm{cell}}}}}}}$ value, which is larger than or equal to the ${k}_{{{{{{\rm{ex}}}}}}}^{{{{{{\rm{in}}}}}}-{{{{{\rm{cell}}}}}}}$ one, is much larger than the ${k}_{{{{{{\rm{open}}}}}}}^{{{{{{\rm{in\; vitro}}}}}}}$ value for G4 and G15 (Fig. 2d, orange shading). Thus, it is concluded that the base pairs involving G4 and G15 open more frequently in living cells than in vitro. That is, the base-pair opening dynamics of the RNA hairpin structure is different in living human cells and in vitro. For the other residues, it is not feasible to determine whether or not the ${k}_{{{{{{\rm{open}}}}}}}^{{{{{{\rm{in}}}}}}-{{{{{\rm{cell}}}}}}}$ values are larger than the ${k}_{{{{{{\rm{open}}}}}}}^{{{{{{\rm{in\; vitro}}}}}}}$ ones, because the black dashed lines reside within the orange shading in Fig. 2d.

### The imino proton exchange rate of the GQ structure in living human cells

**Fig. 3:**
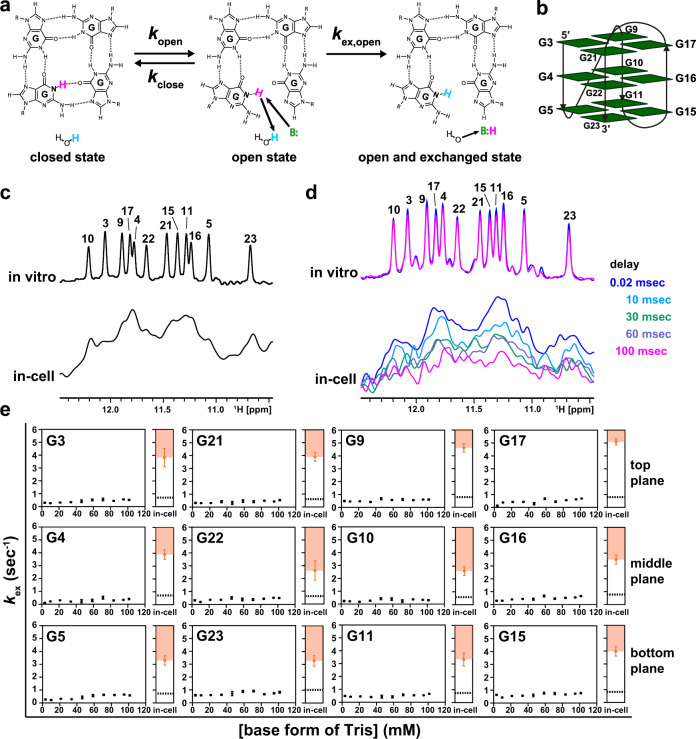
Analysis of the exchange of imino protons of the DNA G-quadruplex structure with the proton of solvent water. **a** Imino proton exchange reaction. **b** Schematics of the G-quadruplex structure of teloDNA^35^. The green boxes indicate guanine bases. **c** 1D ^1^H-NMR spectra of teloDNA recorded under in vitro (upper) and in-cell (lower) conditions. **d** Water magnetization transfer experiments on teloDNA were carried out under in vitro conditions, 300 mM Tris (upper), and under in-cell conditions for living HeLa cells (lower). Spectra recorded with different delay times after water-selective inversion are superimposed. **e** Comparison of the deduced ${k}_{{{{{{\rm{open}}}}}}}$ values of teloDNA between in-cell and in vitro conditions using the ${k}_{{{{{{\rm{ex}}}}}}}$ value as a reference. For each residue, the dashed line indicates the maximum ${k}_{{{{{{\rm{ex}}}}}}}$ value for various concentrations of the base form of Tris. The maximum ${k}_{{{{{{\rm{ex}}}}}}}$ value equals ${k}_{{{{{{\rm{open}}}}}}}^{{{{\rm{in}}}}\; {{{\rm{vitro}}}}}$. The orange shading indicates the deduced ${k}_{{{{{{\rm{open}}}}}}}^{{{{{{\rm{in}}}}}}-{{{{{\rm{cell}}}}}}}$ value range for each residue of teloDNA in HeLa cells. For each base form of Tris concentration, the intensity of imino proton was obtained for each residue (*n* = 1). Standard deviation of the noise signal for the region of 1D ^1^H spectrum in which no signal is present was calculated and used as error bars of intensities. The means and error bars of the ${k}_{{{{{{\rm{ex}}}}}}}$ values were obtained from 50 data sets of intensities constructed by Monte Carlo simulation using the error bars of intensities. Data are presented as mean values ± standard deviation. Source data are provided as a Source Data file.

Next, we investigated the opening/closing dynamics for the base pairs of the GQ structure of a human telomeric DNA (teloDNA: 5′-TTGGG(TTAGGG)_3_A-3′) in living human cells (Fig. 3a). The terminal regions of chromosomes, so-called telomeres, are composed of guanine-rich sequences. These guanine-rich sequences are known to form a GQ structure, which reportedly plays key roles in telomere homeostasis^32–34^. Under in vitro conditions, teloDNA forms the (3+1)-type GQ structure (Fig. 3b)^35^, which comprises three G-quartet planes (Fig. 3a, left and 3b). Each G-quartet plane contains four G-G base-pairs involving hydrogen-bonding of the imino protons (Fig. 3a, left). Here, we aimed to determine the opening/closing dynamics of the G-G base-pairs in the GQ structure and therefore carried out imino proton exchange rate analysis under in vitro and in-cell conditions (Fig. 3a).

Through a water magnetization transfer experiment on the imino protons of teloDNA with various Tris concentrations, the ${k}_{{{{{{\rm{ex}}}}}}}^{{{{{{\rm{in\; vitro}}}}}}}$ value of each G-G base-pair of teloDNA was determined (Fig. 3c, upper and 3d, upper). At high Tris concentrations, ${k}_{{{{{{\rm{ex}}}}}}}^{{{{{{\rm{in\; vitro}}}}}}}$ of all G-G base-pairs reached a plateau, although the change in ${k}_{{{{{{\rm{ex}}}}}}}^{{{{{{\rm{in\; vitro}}}}}}}$ is rather small, and thereby ${k}_{{{{{{\rm{open}}}}}}}^{{{{{{\rm{in\; vitro}}}}}}}$ was determined (Fig. 3e, black dashed line). The ${k}_{{{{{{\rm{open}}}}}}}^{{{{{{\rm{in\; vitro}}}}}}}$ values of all G-G base-pairs turned out to be smaller than those of G-C and A-T base-pairs of duplex structures^36,37^, which indicates that all G-G base-pairs open less frequently that G-C and A-T base-pairs under in vitro conditions.

Before carrying out in-cell NMR experiments, fluorescein-labeled teloDNA (FAM-teloDNA) was introduced into HeLa cells by means of SLO treatment and the intracellular localization of FAM-teloDNA was examined. It was found that almost all FAM-teloDNAs introduced inside HeLa cells were located in the cell nucleus (Supplementary Fig. 3).

Then, in-cell NMR measurements of teloDNA were carried out (Fig. 3c, lower). Imino proton signals were observed in the 10.5-12.5 ppm region in the ^1^H-NMR spectra (Fig. 3c, upper). The pattern of the imino proton signals under in-cell conditions resembled that under in vitro conditions, although the signals under the former conditions were broader (Fig. 3c). This indicates that in living human cells, teloDNA forms a (3+1)-type GQ structure similar to, if not the same as, that formed under in vitro conditions.

A water magnetization transfer experiment on teloDNA introduced into HeLa cells was carried out (Fig. 3d, lower, and Supplementary Fig. 4). The signal intensities at the chemical shift values corresponding to those of individual peaks in the in vitro spectrum were plotted as a function of the delay time (Supplementary Fig. 4), and the imino proton exchange rates, ${k}_{{{{{{\rm{ex}}}}}}}^{{{{{{\rm{in}}}}}}-{{{{{\rm{cell}}}}}}}$, were determined for all G-G base-pairs (Fig. 3e). It turned out that ${k}_{{{{{{\rm{ex}}}}}}}^{{{{{{\rm{in}}}}}}-{{{{{\rm{cell}}}}}}}$ (Fig. 3e, orange dots) is larger than ${k}_{{{{{{\rm{open}}}}}}}^{{{{{{\rm{in\; vitro}}}}}}}$ (Fig. 3e, black dashed lines) for all G-G base-pairs. This indicates that ${k}_{{{{{{\rm{open}}}}}}}^{{{{{{\rm{in}}}}}}-{{{{{\rm{cell}}}}}}}$ is larger than ${k}_{{{{{{\rm{open}}}}}}}^{{{{{{\rm{in\; vitro}}}}}}}$ for all G-G base-pairs, as ${k}_{{{{{{\rm{open}}}}}}}^{{{{{{\rm{in}}}}}}-{{{{{\rm{cell}}}}}}}$ (Fig. 3e, orange shading) is larger than ${k}_{{{{{{\rm{ex}}}}}}}^{{{{{{\rm{in}}}}}}-{{{{{\rm{cell}}}}}}}$ (Fig. 3e, orange dots, Eq. (2)). This indicates that all G-G base-pairs in the GQ structure of teloDNA open more frequently in living human cells than in vitro.

Imino proton signals under in-cell conditions are very broad and thus overlapping (Fig. 3d, lower). Therefore, we tried an alternative analysis. The average of the intensities for twelve peaks was used to determine the average ${k}_{{{{{{\rm{ex}}}}}}}$ values for both in vitro and in-cell conditions (Supplementary Fig. 5). Our claim that ${k}_{{{{{{\rm{open}}}}}}}^{{{{{{\rm{in}}}}}}-{{{{{\rm{cell}}}}}}}$ is higher than ${k}_{{{{{{\rm{open}}}}}}}^{{{{{{\rm{in\; vitro}}}}}}}$ is confirmed, even if the average ${k}_{{{{{{\rm{ex}}}}}}}$ values are used. Additionally, imino proton signals under in-cell conditions are also noisy (Fig. 3d, lower). The effect of the noise was estimated by obtaining the error bars of the ${k}_{{{{{{\rm{ex}}}}}}}$ values from the data sets constructed by Monte Carlo simulation using the noise level (see the Methods section for detail). It turned out that although the error bars of the ${k}_{{{{{{\rm{ex}}}}}}}$ values under in-cell conditions are larger due to the noise than those under in vitro conditions, our claim that ${k}_{{{{{{\rm{open}}}}}}}^{{{{{{\rm{in}}}}}}-{{{{{\rm{cell}}}}}}}$ is higher than ${k}_{{{{{{\rm{open}}}}}}}^{{{{{{\rm{in\; vitro}}}}}}}$ still holds (Fig. 3e).

### The imino proton exchange rate of the hairpin structure in the presence of various crowding agents

**Fig. 4:**
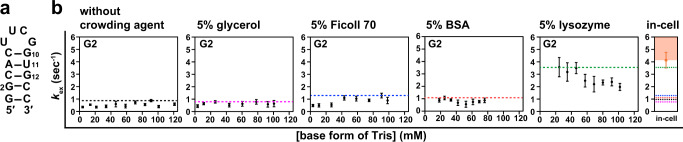
Analysis of the exchange of imino protons of the RNA hairpin structure in the presence of crowding agents. **a** Secondary structure of fully 2′-*O*-methylated hpRNA14. **b** Comparison of ${k}_{{{{{{\rm{open}}}}}}}$ values for G2 of hpRNA14 between in-cell conditions and in vitro conditions in either the absence or presence of a crowding agent (glycerol, Ficoll, BSA, and lysozyme). The dashed lines indicate the maximum ${k}_{{{{{{\rm{ex}}}}}}}$ values for G2 obtained under in vitro conditions in the absence (black) and presence of either glycerol (pink), Ficoll (blue), BSA (red), or lysozyme (green). Each maximum ${k}_{{{{{{\rm{ex}}}}}}}$ value equals ${k}_{{{{{{\rm{open}}}}}}}$ under individual conditions. The orange shading indicates the deduced ${k}_{{{{{{\rm{open}}}}}}}^{{{{{{\rm{in}}}}}}-{{{{{\rm{cell}}}}}}}$ value range for G2 of hpRNA14 in HeLa cells. For each base form of Tris concentration, the intensity of imino proton was obtained for each residue (*n* = 1). Standard deviation of the noise signal for the region of 1D ^1^H spectrum in which no signal is present was calculated and used as error bars of intensities under individual conditions. The means and error bars of the ${k}_{{{{{{\rm{ex}}}}}}}$ values were obtained from 50 data sets of intensities constructed by Monte Carlo simulation using the error bars of intensities. Data are presented as mean values ± standard deviation. Source data are provided as a Source Data file.

To determine the origin of the observation that at least for some residues of hpRNA20, ${k}_{{{{{{\rm{open}}}}}}}$ is larger under in-cell conditions than under in vitro conditions, the imino proton exchange rates were determined for an RNA hairpin structure in the presence of various crowding agents that reportedly mimic cellular conditions, e.g., glycerol, Ficoll PM70 (Ficoll), bovine serum albumin (BSA), and lysozyme^3,38^. Glycerol and Ficoll are crowding agents that are often used to examine the effect of changes in water activity and the excluded volume effect, respectively. To assess the interaction with endogenous proteins, BSA and lysozyme were used as representatives of proteins that are negatively and positively charged, respectively. It was found that hpRNA20 precipitates upon the addition of lysozyme. Therefore, here, we used a hairpin RNA, hpRNA14 (Fig. 4a), that has the same sequence as hpRNA20 but lacks the terminal three base pairs, because hpRNA14 does not precipitate upon the addition of lysozyme. hpRNA14 was used previously in our in-cell NMR study^13^. Isolated imino proton signals were observed for G2 and U11 of hpRNA14, while the peaks of G10 and G12 were overlapping. Therefore, we analyzed the imino proton peaks of G2 and U11.

It turned out that ${k}_{{{{{{\rm{ex}}}}}}}^{{{{{{\rm{in}}}}}}-{{{{{\rm{cell}}}}}}}$ (Fig. 4b, orange dot) is larger than ${k}_{{{{{{\rm{open}}}}}}}^{{{{{{\rm{in\; vitro}}}}}}}$ (Fig. 4b, black dashed line) for a base pair involving G2. This indicates that ${k}_{{{{{{\rm{open}}}}}}}^{{{{{{\rm{in}}}}}}-{{{{{\rm{cell}}}}}}}$ is larger than ${k}_{{{{{{\rm{open}}}}}}}^{{{{{{\rm{in\; vitro}}}}}}}$ for the base pair involving G2, as ${k}_{{{{{{\rm{open}}}}}}}^{{{{{{\rm{in}}}}}}-{{{{{\rm{cell}}}}}}}$ (Fig. 4b, orange shading) is larger than or equal to ${k}_{{{{{{\rm{ex}}}}}}}^{{{{{{\rm{in}}}}}}-{{{{{\rm{cell}}}}}}}$ (Fig. 4b, orange dot), as explained above. This indicates that the base pair involving G2 of hpRNA14 opens more frequently in living human cells than in vitro, as was observed for G4 and G15 of hpRNA20. For a base pair involving U11, it was not feasible to determine whether or not the ${k}_{{{{{{\rm{open}}}}}}}^{{{{{{\rm{in}}}}}}-{{{{{\rm{cell}}}}}}}$ value is larger than the ${k}_{{{{{{\rm{open}}}}}}}^{{{{{{\rm{in\; vitro}}}}}}}$ one, because the maximum value of ${k}_{{{{{{\rm{ex}}}}}}}^{{{{{{\rm{in\; vitro}}}}}}}$ (36.1 ± 1.1 s^−1^) for various Tris concentrations turned out to be larger than the ${k}_{{{{{{\rm{ex}}}}}}}^{{{{{{\rm{in}}}}}}-{{{{{\rm{cell}}}}}}}$ (5.6 ± 1.1 s^−1^), as was observed for U14, U18, and G19 of hpRNA20. Therefore, the effects of crowding agents were examined for G2 of hpRNA14.

In the presence of either glycerol or Ficoll, ${k}_{{{{{{\rm{ex}}}}}}}^{{{{{{\rm{in\; vitro}}}}}}\,({{{{{\rm{glycerol}}}}}})}$ or ${k}_{{{{{{\rm{ex}}}}}}}^{{{{{{\rm{in\; vitro}}}}}}\,({{{{{\rm{Ficoll}}}}}})}$ of G2 was measured at various Tris concentrations (Fig. 4b). The maximum value of ${k}_{{{{{{\rm{ex}}}}}}}$, which corresponds to ${k}_{{{{{{\rm{open}}}}}}}$, in the presence of either glycerol or Ficoll was almost the same as that without any crowding agent. These results indicated that a change in water activity and the excluded volume effect may not be a dominant origin of the observation that ${k}_{{{{{{\rm{open}}}}}}}$ is larger under in-cell conditions than under in vitro ones.

Next, in the presence of either BSA or lysozyme, ${k}_{{{{{{\rm{ex}}}}}}}^{{{{{{\rm{in\; vitro}}}}}}\,({{{{{\rm{BSA}}}}}})}$ or ${k}_{{{{{{\rm{ex}}}}}}}^{{{{{{\rm{in\; vitro}}}}}}\,({{{{{\rm{lysozyme}}}}}})}$ of G2 was measured at various Tris concentrations (Fig. 4b). The maximum value of ${k}_{{{{{{\rm{ex}}}}}}}$, which corresponds to ${k}_{{{{{{\rm{open}}}}}}}$, in the presence of BSA (Fig. 4b, red dashed line) was almost the same as that without any crowding agent (Fig. 4b, black dashed line). In contrast, the maximum value of ${k}_{{{{{{\rm{ex}}}}}}}^{{{{{{\rm{in\; vitro}}}}}}\,({{{{{\rm{lysozyme}}}}}})}$ was larger than that without any crowding agent (Fig. 4b, black dashed line). This indicates that ${k}_{{{{{{\rm{open}}}}}}}^{{{{{{\rm{in\; vitro}}}}}}\,({{{{{\rm{lysozyme}}}}}})}$ is larger than ${k}_{{{{{{\rm{open}}}}}}}^{{{{{{\rm{in\; vitro}}}}}}}$. It was noticed that ${k}_{{{{{{\rm{open}}}}}}}^{{{{{{\rm{in\; vitro}}}}}}\,({{{{{\rm{lysozyme}}}}}})}$ is almost the same as ${k}_{{{{{{\rm{ex}}}}}}}^{{{{{{\rm{in}}}}}}-{{{{{\rm{cell}}}}}}}$, which is the lower limit of ${k}_{{{{{{\rm{open}}}}}}}^{{{{{{\rm{in}}}}}}-{{{{{\rm{cell}}}}}}}$. These results indicate that the interaction between hpRNA14 and lysozyme promotes the base-pair opening of hpRNA14. BSA and lysozyme are negatively and positively charged, respectively. Because hpRNA14 is negatively charged, lysozyme, but not BSA, electrostatically interacts with hpRNA14, which could result in promotion of the base-pair opening. These results suggest that the interaction between the hairpin structure of RNA and endogenous proteins having a net positive charge may be a dominant origin of the observation that ${k}_{{{{{{\rm{open}}}}}}}$ is larger under in-cell conditions than under in vitro ones.

### The imino proton exchange rate of the GQ structure in the presence of various crowding agents

**Fig. 5:**
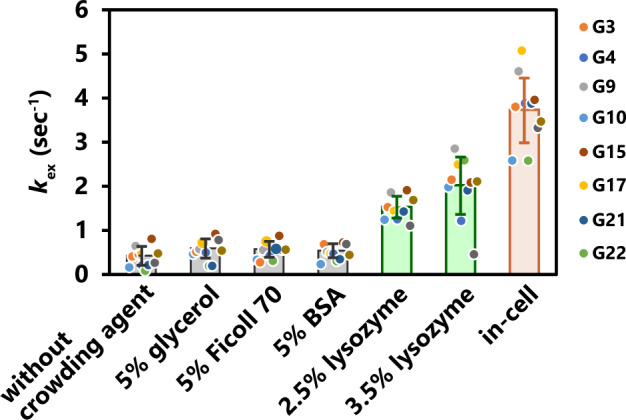
Analysis of the exchange of imino protons of the DNA GQ structure in the presence of crowding agents. The ${k}_{{{{{{\rm{ex}}}}}}}$ values of each guanine in teloDNA (excluding G5 and G23) were obtained under in vitro conditions in the absence and presence of a crowding agent (glycerol, Ficoll, BSA, and lysozyme) at 300 mM Tris. The ${k}_{{{{{{\rm{ex}}}}}}}$ values at 300 mM Tris can be regarded to be equal to ${k}_{{{{{{\rm{open}}}}}}}$ under individual in vitro conditions (see text for details). The intensity of imino proton was obtained for each residue (*n* = 1). Standard deviation of the noise signal for the region of 1D ^1^H spectrum in which no signal is present was calculated and used as error bars of intensities. The mean of the ${k}_{{{{{{\rm{ex}}}}}}}$ values was obtained from 50 data sets of intensities constructed by Monte Carlo simulation using the error bars of intensities for each residue. The obtained means were averaged over residues under individual conditions. The averaged ${k}_{{{{{{\rm{ex}}}}}}}$ values under individual in vitro and in-cell conditions are indicated. The error bars are the standard deviation. Source data are provided as a Source Data file.

To determine the origin of the observation that for all residues of teloDNA, ${k}_{{{{{{\rm{open}}}}}}}$ is larger under in-cell conditions than under in vitro ones, analysis with crowding agents was carried out also for the GQ structure of teloDNA. Figure 3e indicates that ${k}_{{{{{{\rm{ex}}}}}}}$ seems to reach the maximum around at 100 mM base form of Tris, although the change in ${k}_{{{{{{\rm{ex}}}}}}}^{{{{{{\rm{in\; vitro}}}}}}}$ is small. 100 mM base form of Tris corresponds to ca. 300 mM total concentration of Tris. Therefore, it is supposed that ${k}_{{{{{{\rm{ex}}}}}}}$ at 300 mM total concentration of Tris equals ${k}_{{{{{{\rm{open}}}}}}}$. Thus, the analysis with crowding agents was carried out with the total concentration of 300 mM Tris to obtain the ${k}_{{{{{{\rm{open}}}}}}}$ value. The ${k}_{{{{{{\rm{ex}}}}}}}$ of all residues of teloDNA except for G5 and G23, whose imino proton signals overlapped with those of the BSA and lysozyme, was obtained in the presence of each crowding agent. For each crowding agent, the ${k}_{{{{{{\rm{ex}}}}}}}$ value turned out to be similar for all residues. Therefore, the ${k}_{{{{{{\rm{ex}}}}}}}$ values of all the residues obtained for each crowding agent were averaged over residues (Fig. 5).

The addition of either glycerol, Ficoll, or BSA in the presence of 300 mM Tris hardly changed ${k}_{{{{{{\rm{ex}}}}}}}$, which is regarded to be equal to ${k}_{{{{{{\rm{open}}}}}}}$, as explained above (Fig. 5). On the other hand, the addition of lysozyme in the presence of 300 mM Tris did change ${k}_{{{{{{\rm{ex}}}}}}}$ (= ${k}_{{{{{{\rm{open}}}}}}}$) dose-dependently (Fig. 5). The ${k}_{{{{{{\rm{ex}}}}}}}$ value (= ${k}_{{{{{{\rm{open}}}}}}}$) in the presence of 3.5 % lysozyme approached the ${k}_{{{{{{\rm{ex}}}}}}}$ value under in-cell conditions, which is the lower limit of ${k}_{{{{{{\rm{open}}}}}}}$ under in-cell conditions. These results suggest that also for the GQ structure of teloDNA, the interaction with endogenous proteins having a net positive charge is the dominant origin of the observation of the larger ${k}_{{{{{{\rm{open}}}}}}}$ under in-cell conditions than under in vitro ones.

## Discussion

In the present study, we investigated the base-pair opening dynamics of RNA hairpin and DNA GQ structures in living human cells by means of the in-cell NMR technique combined with imino proton exchange rate analysis. The imino proton exchange rate, ${k}_{{{{{{\rm{ex}}}}}}}^{{{{{{\rm{in}}}}}}-{{{{{\rm{cell}}}}}}}$, of each base pair was successfully determined, and the ranges of the rate constants for base-pair opening, ${k}_{{{{{{\rm{open}}}}}}}^{{{{{{\rm{in}}}}}}-{{{{{\rm{cell}}}}}}}$, were determined in living human cells, which has not been reported before.

We recorded the 1D ^1^H in-cell NMR spectra before the first data point and after the very last point of the in-cell water magnetization transfer experiments. In the case of hpRNA20, superimposition of these two spectra shown in Supplementary Fig. 6a indicated that the intensities of all the imino proton signals are retained. In our previous in-cell NMR studies^13^, the bioreactor system^19,39,40^ was not used. In that case, the leakage of oligonucleotides was observed. In the current study, we used bioreactor system. This drastically improved the cell viability, which resulted in almost no leakage of oligonucleotides retaining the intensities of all the imino proton signals. The retention of the intensities of all the imino proton signals also revealed that the effect of the endogenous nuclease is negligible.

The spectra before and after the in-cell water magnetization transfer experiments for teloDNA are superimposed in Supplementary Fig. 6b. The intensities of the imino proton signals are again basically retained for the most residues. For some residues, however, slight reduction in intensities is seen. Maximum reduction is observed for G4 and G17 residues, ca. 16%. Accordingly, we employed the correction to the data. As we recorded five water magnetization transfer spectra with five different delay times, the intensities of the corresponding imino proton signals of the spectrum recorded second, third, fourth, and fifth were corrected by the factors of 1/0.96, 1/0.92, 1/0.88, and 1/0.84, respectively. Then, ${k}_{{{{{{\rm{ex}}}}}}}$ values were obtained (Supplementary Fig. 6c, d). The ${k}_{{{{{{\rm{ex}}}}}}}$ values with and without the corrections turn out to be the same within the error bars caused by the noise present in the spectra. This analysis confirmed that the artificial effects on ${k}_{{{{{{\rm{ex}}}}}}}$ values caused by either leakage or endogenous nuclease are negligible for teloDNA as well.

As is summarized in the paper of Hwang et al.^41^, artifacts due to other magnetization transfer mechanisms such as (i) NOEs from the protons that have chemical shifts coincident with water or (ii) exchange-relayed NOEs from rapidly exchanging protons (hydroxyl or amine groups) may have affected our study. However, the effects of these mechanisms would not be significant in our study, as indicated by following analyses:

Firstly, we confirmed that the mechanism (ii) above is unlikely in our study as shown below. We prepared two samples: 20 µM teloDNA, 2.5% lysozyme, 115 mM CH_3_COOK, 2.5 mM MgCl_2_, and 300 mM of total concentration of Tris dissolved in either (1) D_2_O/H_2_O = 10%/90% or (2) D_2_O/H_2_O = 50%/50%. We carried out the water magnetization transfer experiments using these two samples, in which the densities of water protons are different with respect to each other. We carried out two measurements for each sample. In one measurement, the selective inversion pulse was applied at 4.7 ppm (the chemical shift for water), and then after 100 ms delay time, imino-proton spectrum was recorded. In the other measurement, the selective inversion pulse was applied at −30 ppm (no signals present) instead. The relative intensity of the imino-proton signals was obtained by dividing the intensity of the imino-proton signals of the former measurement by that of the latter measurement (Supplementary Fig. 7). It turned out that the relative intensity of the imino-proton signals was the same for both the samples within error bars. As the water proton density is higher for sample (1) than sample (2), the effect of exchange-relayed NOEs could be more pronounced for sample (1) and thus the relative intensity of the imino proton signals should be smaller for sample (1) than sample (2). The observation of the same relative intensity for sample (1) and sample (2) reveals that the effect of exchange-relayed NOEs is rather moderate, if any.

Secondly, we supposed that the mechanism (i) above is also unlikely as shown below. The Q3 pulse was used for selective inversion of water. Simulation by TopSpin 3.6.2 indicated that the inversion efficiency is 100% for the region $\pm$0.25 ppm of water position and drops to 50% at $\pm$0.45 ppm of water position. The protons located in $\pm$0.45 ppm region are H3′s and roughly a half of H4′s. These protons could be “the protons that have chemical shifts coincident with water”. The structure of teloDNA used in our in-cell NMR study had been determined^35^ (PDB ID: 2GKU). We investigated the distances between imino protons and either H3′s or H4′s for twelve deposited structures. For the identification of the short distances, we put the criteria to satisfy that the certain proton-proton distance is shorter than the certain distance for more than six structures out of the twelve structures. There was no H3′ closer than 5 Å from imino protons. There were only two H4′s closer than 5 Å from imino protons. This analysis suggests that the mechanism (i) above is not so likely. The distance between H4′ of the T13 residue and imino proton of the G11 residue, and the distance between H4′ of the A14 residue and imino proton of the G15 residue are smaller than 5 Å. However, imino protons of the G11 and G15 residues do not particularly exhibit the larger ${k}_{{{{{{\rm{ex}}}}}}}^{{{{{{\rm{in}}}}}}-{{{{{\rm{cell}}}}}}}$ values compared to the other imino protons (Fig. 3e). This also suggests that the effect of the mechanism (i) is moderate, if any. Furthermore, we used relatively short delay time (<100 ms) after the selective inversion pulse for the proton of solvent water. This could also reduce the effect of the mechanism (i) including the effect of the spin diffusion. Thus, although it is hard to completely exclude the possible effect of the mechanism (i), we assume the effect is tolerable.

There is also indirect support to our idea. When the amount of lysozyme as a crowding reagent was increased under in vitro conditions, the corresponding ${k}_{{{{{{\rm{ex}}}}}}}$ value increased, approaching the ${k}_{{{{{{\rm{ex}}}}}}}$ value obtained under in-cell conditions (Fig. 5). On the other hand, the full width of half height of imino proton signals increased just slightly when the amount of lysozyme was increased under in vitro conditions (Supplementary Fig. 8). In fact, the full width of half height for 3.5% lysozyme and 5% glycerol are the same within error bars (Supplementary Fig. 8), although the ${k}_{{{{{{\rm{ex}}}}}}}$ value for 3.5% lysozyme is ca. four times larger than that for 5% glycerol (Fig. 5). As the full width of half height is similar for 3.5% lysozyme and 5% glycerol, a rotational correlation time should be similar for 3.5% lysozyme and 5% glycerol and thus the effect of NOE and spin diffusion should also be similar, if they are. Therefore, the four-times increase in the ${k}_{{{{{{\rm{ex}}}}}}}$ value for 3.5% lysozyme are supposed to be due to increase in chemical exchange of imino protons with water protons caused by 3.5% lysozyme. The ${k}_{{{{{{\rm{ex}}}}}}}$ value for 3.5% lysozyme under in vitro conditions already reached more than half of the ${k}_{{{{{{\rm{ex}}}}}}}$ value obtained under in-cell conditions, these two ${k}_{{{{{{\rm{ex}}}}}}}$ values becoming comparable on a broader basis (Fig. 5). This implies that the increase in the ${k}_{{{{{{\rm{ex}}}}}}}$ value observed for in-cell conditions would also be mainly due to chemical exchange of imino protons with water protons.

**Fig. 6:**
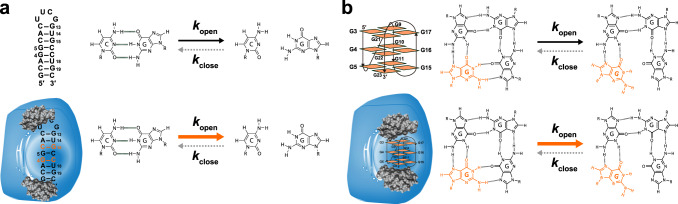
Proposed model of base-pair opening dynamics in living cells. **a** (hpRNA20) The base pairs involving G4 and G15 located at the center of the stem region open not so frequently under in vitro conditions. On the other hand, the base pairs open more frequently in living cells (indicated by bold orange arrow). **b** (teloDNA) All G-G base-pairs open not so frequently under in vitro conditions. On the other hand, all the G-G base-pairs open more frequently in living cells (indicated by bold orange arrow).

Among the base pairs of hpRNA20, the G-C base-pairs involving G4 and G15, which are located away from the ends of the stem structure, were able to be used to evaluate the ${k}_{{{{{{\rm{open}}}}}}}$ values of the base pairs obtained under in-cell conditions (${k}_{{{{{{\rm{open}}}}}}}^{{{{{{\rm{in}}}}}}-{{{{{\rm{cell}}}}}}}$). Firstly, the ${k}_{{{{{{\rm{open}}}}}}}$ values of the base pairs involving G4 and G15 obtained under in vitro conditions (${k}_{{{{{{\rm{open}}}}}}}^{{{{{{\rm{in\; vitro}}}}}}}$) were shown to be lower than those involving U14, U18, and G19, which are located at the ends of the stem structure (Table 1), indicating base-pair opening is suppressed in the central region. Then it turned out that the ${k}_{{{{{{\rm{open}}}}}}}^{{{{{{\rm{in}}}}}}-{{{{{\rm{cell}}}}}}}$ values are much larger than the ${k}_{{{{{{\rm{open}}}}}}}^{{{{{{\rm{in\; vitro}}}}}}}$ ones for the base pairs involving G4 and G15 (Fig. 2d, orange shading), indicating that the base pairs in the central region of the stem structure open more frequently inside living cells (Fig. 6). Although, it was not possible to determine whether or not the ${k}_{{{{{{\rm{open}}}}}}}^{{{{{{\rm{in}}}}}}-{{{{{\rm{cell}}}}}}}$ values are larger than the ${k}_{{{{{{\rm{open}}}}}}}^{{{{{{\rm{in\; vitro}}}}}}}$ ones for the base pairs involving U14, U18, and G19, it is likely that the frequency of opening of these base pairs is also increased under in-cell conditions.

In the case of teloDNA, all twelve guanines involved in the G-G base-pair formation were used to determine the ${k}_{{{{{{\rm{open}}}}}}}^{{{{{{\rm{in}}}}}}-{{{{{\rm{cell}}}}}}}$ values. It turned out that the ${k}_{{{{{{\rm{open}}}}}}}^{{{{{{\rm{in}}}}}}-{{{{{\rm{cell}}}}}}}$ values are larger than the ${k}_{{{{{{\rm{open}}}}}}}^{{{{{{\rm{in\; vitro}}}}}}}$ ones for all twelve guanines (Fig. 3e), indicating that all G-G base pairs of the GQ structure open more frequently under in-cell conditions than in vitro ones (Fig. 6). This increase in the frequency of the G-G base-pair opening may suggest an increase in the frequency of partial unfolding of the GQ structure.

Evaluation of the ${k}_{{{{{{\rm{open}}}}}}}^{{{{{{\rm{in}}}}}}-{{{{{\rm{cell}}}}}}}$ values suggested that at least some G-C base pairs of the hairpin structure and all G-G base-pairs of the GQ structure open more frequently in living human cells than in vitro (Figs. 2d, 3e). Then, analysis using crowding reagents showed that the electrostatic interactions with endogenous proteins could be a key factor for the increase in frequency of base-pair opening (Fig. 6). Recently, weak and transient interactions between proteins in an intracellular environment, collectively termed quinary interactions, were shown to play important roles inside cells in various biological events, e.g., signal transduction, gene regulation, and stress adaptation^10,42–47^. Additionally, quinary interactions have also been shown to determine the structural stability of proteins inside cells^48^. However, the effect of quinary interactions on the structural stability and dynamics of functional DNAs and RNAs has not been reported. This study provides an experimental approach for understanding of the effect of quinary interactions on the base-pair opening dynamics of nucleic acids.

GQ structures are reportedly formed not only in telomeres but also in the promoter regions of some genes, replication origins, and 5′- and/or 3′-untranslated regions of some mRNAs, and thereby are regarded as important gene regulators^32–34,49–51^. It is thought that the GQ structures are not static but undergo partial structural changes inside cells. Our study may suggest that the aforementioned quinary interactions play important roles in structural changes of GQ structures related to the functions inside cells. It was reported on the basis of the single-molecule fluorescence imaging and time-dependent chemical trapping of unfolded GQs that GQs fluctuate between folded and unfolded states inside the cells^52^. This is consistent with our finding revealed at atomic resolution.

Recently, DNA GQ structures formed in the telomere and promoter regions of oncogenes, such as *c-myc*, are considered important targets for anti-cancer drugs, and therefore the development of small compounds targeting such GQ structures became an urgent task^53–56^. RNA tertiary structures are also considered important targets for drugs against neurodegenerative diseases^57^ and infectious bacterial diseases^58^. Our current study may suggest that in addition to closed forms, partially unfolded structures that are supposed to be present inside cells can also be targets for drugs. In this context, our in-cell NMR technique can be applied to develop drugs against DNA and RNA targets.

## Methods

### Oligonucleotide preparation

The fully 2′-*O*-methylated oligoribonucleotides, hpRNA20 (5′-GCAGGCACUUCGGUGCCUGC-3′) and hpRNA14 (5′-GGCACUUCGGUGCC-3′), were synthesized, purified, and de-salted by Hokkaido System Science Co., Ltd. (Hokkaido, Japan). The oligodeoxyribonucleotides, teloDNA (5′-TTGGG(TTAGGG)_3_A-3′), and that with a 5′-fluorescein (FAM)-label, were synthesized, purified, and de-salted by FASMAC Co., Ltd. (Kanagawa, Japan).

### In vitro NMR measurements

Each oligonucleotide was dissolved in transport buffer (TB: 25 mM HEPES-KOH (pH 7.0), 115 mM CH_3_COOK, and 2.5 mM MgCl_2_) containing 10% D_2_O and 20 µM 4,4-dimethyl-4-silapentane-1-sulfonic acid (DSS) to a final concentration of 150 µM. The solution was heated at 95 °C for 5 min and then cooled down to 30 °C at the rate of 1 °C/min. 1D ^1^H NMR spectra were acquired at 18 °C by the band-selective optimized-flip-angle short-transient (SOFAST)^59^ technique with the band-selective excitation PC9 and refocusing r-SNOB pulses centered at the imino proton region. Briefly, the pulse sequence was made by modifying the 1D ^1^H HET-SOFAST experiment reported by Schanda et al.^60^. We omitted the HET pulse scheme from the 1D ^1^H HET-SOFAST experiment to record 1D ^1^H NMR spectra. All in vitro and in-cell NMR measurements were carried out using a Bruker BioSpin AVANCE III HD 600 spectrometer (Bruker, Billerica, MA, USA) equipped with a cryogenic probe. All in vitro and in-cell NMR data were processed and analyzed with TOPSPIN 3.6.2 (Bruker, Billerica, MA, USA).

### Oligonucleotide introduction into HeLa cells by streptolysin O (SLO) treatment

Each oligonucleotide was introduced into HeLa cells by SLO treatment^13^. $4\times {10}^{7}$ HeLa cells in ice-cold phosphate-buffered saline (PBS) were mixed with a final concentration of 0.06 μg/mL SLO (BioAcademia, Osaka, Japan; catalog code 01-531) and incubated at 4 °C for 10 min. The collected cells were washed three times with ice-cold PBS. Then the cells were resuspended in TB, incubated at 32 °C for another 5 min, and then transferred to TB that contains cytosol^29^ prepared from mouse liver cells, an ATP regenerating system (5 mM ATP, 50 ng/mL creatine kinase, and 2.62 mg/mL creatine phosphate), 1 mg/mL glucose, 1 mM GTP, and 1 mM oligonucleotide of interest. After incubation at 32 °C for 30 min with gentle shaking, the SLO-mediated pores were resealed by addition of CaCl_2_ to a final concentration of 1 mM and subsequent incubation at 32 °C for 5 min with gentle shaking. The cells were then washed three times with 10 mL of Leibovitz’s L-15 medium containing 1 mM CaCl_2_.

### In-cell NMR measurements using a bioreactor system

In-cell NMR measurements were carried out using a bioreactor system^19,39,40^. A cell suspension of HeLa cells containing the oligonucleotide of interest was mixed with the same volume of 0.9 x Leibovitz’s L-15 medium containing 3% of low-melting-temperature agarose (Lonza), 10% D_2_O, and 20 µM DSS, which was prewarmed at 37 °C. The mixture was transferred to a Teflon tube (inner diameter of 0.5 mm) and chilled on ice. The resulting thread-like agarose gel containing cells was transferred to a 5 mm NMR tube that was connected to the bioreactor system. 0.9 x Leibovitz’s L-15 medium containing 10% D_2_O and 20 µM DSS was supplied at a flow rate of 100 µL/min.

1D ^1^H NMR spectra were acquired at 18 °C by the SOFAST technique as described above. The numbers of scans were 1024 and 2048 for hairpin RNAs and teloDNA, respectively.

### Imino proton exchange rate analysis under in vitro and in-cell conditions

Each oligonucleotide was dissolved in Tris-HCl buffer containing CH_3_COOK, MgCl_2_, D_2_O, and DSS. The solution was heated and cooled as described above. Then, either glycerol, Ficoll PM70 (Cytiva), bovine serum albumin (BSA), or lysozyme was added as a crowding agent. Then, the pH was adjusted to within the range of 7.9 and 8.2 for hpRNA14 and hpRNA20, and 8.2 and 8.5 for teloDNA. The final sample contained 150 µM oligonucleotide, 115 mM CH_3_COOK, 2.5 mM MgCl_2_, 10% D_2_O, 20 µM DSS, various total concentrations of Tris, and either 0, 2.5, 3.5, or 5% crowding agent. For the experiments involving teloDNA in the presence of lysozyme, the DNA concentration was 20 µM to avoid precipitation. The concentration of the active form of a Tris base catalyst, [base form of Tris], was calculated using the following equation^25^.

$$\left[{{{{{\rm{base}}}}}}\; {{{{{\rm{form}}}}}}\; {{{{{\rm{of}}}}}}\; {{{{{\rm{Tris}}}}}}\right]={c}_{0} \cdot \frac{{10}^{-{{{{{\rm{p}}}}}}K{{{{{\rm{a}}}}}}}}{{10}^{-{{{{{\rm{pH}}}}}}}+{10}^{-{{{{{\rm{p}}}}}}K{{{{{\rm{a}}}}}}}}$$

where, *c*_0_ is the total concentration of Tris and *K*a is the acid dissociation constant of Tris.

To avoid changes of the pH and concentrations of crowding agents during the Tris titration experiment, a titration experiment was carried out utilizing two 300 µL NMR samples. At the starting point of titration, samples containing 10 mM Tris (sample 1) and 300 mM Tris (sample 2) were prepared. For each point in the titration, equal volumes of the samples were exchanged. By doing this, the Tris concentration increased for sample 1 and decreased for sample 2, while the pH and concentrations of crowding agents remained the same. Samples 1 and 2 of hpRNA14 for BSA and lysozyme contained 60 mM and 250 mM Tris, respectively, to avoid precipitation.

The longitudinal relaxation rate constant of the imino proton (*R*_1a_ = 1/*T*_1_) was determined by means of a selective inversion recovery experiment on the imino protons at 18 °C under in vitro and in-cell conditions. The selective inversion of imino protons was carried out with a Q3 pulse covering a bandwidth of 5 ppm. A Q3 pulse centered at 13 and 11.5 ppm was used for hairpin RNAs and teloDNA, respectively. The imino-selective excitation following the Q3 pulse was carried out by the SOFAST technique with PC9 and rSNOB pulses. The longitudinal relaxation rate constant of water (*R*_1w_) was determined by means of a saturation recovery experiment^25^ under in vitro and in-cell conditions. The imino proton exchange rates (${k}_{{{{{{\rm{ex}}}}}}}$) was determined through a water magnetization transfer experiment at 18 °C under in vitro and in-cell conditions. The selective inversion of water protons was carried out with a Q3 pulse. The imino-selective excitation following the Q3 pulse and various delays was carried out by the SOFAST technique with PC9 and rSNOB pulses. The PC9 and rSNOB pulses were set at the center of imino proton regions, i.e., at 13 and 11.5 ppm for hairpin RNAs and teloDNA, respectively. The experiments were carried out in “scrambled order” of delays, in which the spectra with delay times of 0.1, 0.001, 0.01, 0.06, and 0.03 s were recorded in this order. The exchange rates were obtained by fitting to the following equation^25^

3 $$\frac{{I}_{(t)}}{{I}_{0}}=1-2\frac{{k}_{{{{{{\rm{ex}}}}}}}}{({R}_{1{{{{{\rm{w}}}}}}}-{R}_{1{{{{{\rm{a}}}}}}})}({e}^{-{R}_{1{{{{{\rm{a}}}}}}}t}-{e}^{-{R}_{1{{{{{\rm{w}}}}}}}t})$$

where *I*_0_ and *I*_(t)_ are the signal intensities of the imino proton in the water magnetization transfer experiment at delay time zero and *t*, respectively. Standard deviation of the noise signal for the region of 1D ^1^H spectrum in which no signal is present was calculated and used as error bars of intensities. The error bars of the ${k}_{{{{{{\rm{ex}}}}}}}$ values were obtained from 50 data sets constructed by Monte Carlo simulation using the error bars.

### Confocal fluorescence microscopy analysis

SLO-treated HeLa cells were suspended in PBS containing 8 µM Hoechst 33342. After 20 min incubation at room temperature, images were acquired with an Olympus FV1000 confocal scanning laser microscope equipped with a 60× UPlanSApo objective.

### Reporting summary

Further information on research design is available in the Nature Portfolio Reporting Summary linked to this article.

## Supplementary information

Supplementary Information

## Data Availability

The data that support this study are available from the corresponding author upon reasonable request. The structure of teloDNA used in our in-cell NMR study was obtained from the Protein Data Bank PDB ID 2GKU. Source data are available for Figs. 2–5 and Supplementary Figs. 2, and 4–8. Source data are provided with this paper.

## Source data

Source Data
Reporting Summary
