# Supplementary material for: Shedding light on the base-pair opening dynamics of nucleic acids in living human cells
Source: Nat Commun. 2022 Nov 29;13:7143. doi: 10.1038/s41467-022-34822-4 (PMC9708698; doi:10.1038/s41467-022-34822-4)
Supplement: Supplementary file 1 — Supplementary Information [file 41467_2022_34822_MOESM1_ESM.pdf]

Supplementary Information

Shedding light on the base-pair opening dynamics of nucleic acids in living human cells

Yudai Yamaoki[1,2,3], Takashi Nagata[1,2,3,*], Keiko Kondo[1,3,4], Tomoki Sakamoto[2], Shohei Takami[2] & Masato Katahira[1,2,3,4,*]

[1]Institute of Advanced Energy, Kyoto University, Uji, Kyoto 611-0011, Japan

[2]Graduate School of Energy Science, Kyoto University, Uji, Kyoto 611-0011, Japan

[3]Integrated Research Center for Carbon Negative Science, Institute of Advanced Energy, Kyoto University, Uji, 611-0011, Japan

[4]Biomass Product Tree Industry-Academia Collaborative Research Laboratory, Kyoto University, Uji, Kyoto 611-0011, Japan

*Corresponding authors

E-mail: katahira.masato.6u@kyoto-u.ac.jp,

nagata.takashi.6w@kyoto-u.ac.jp

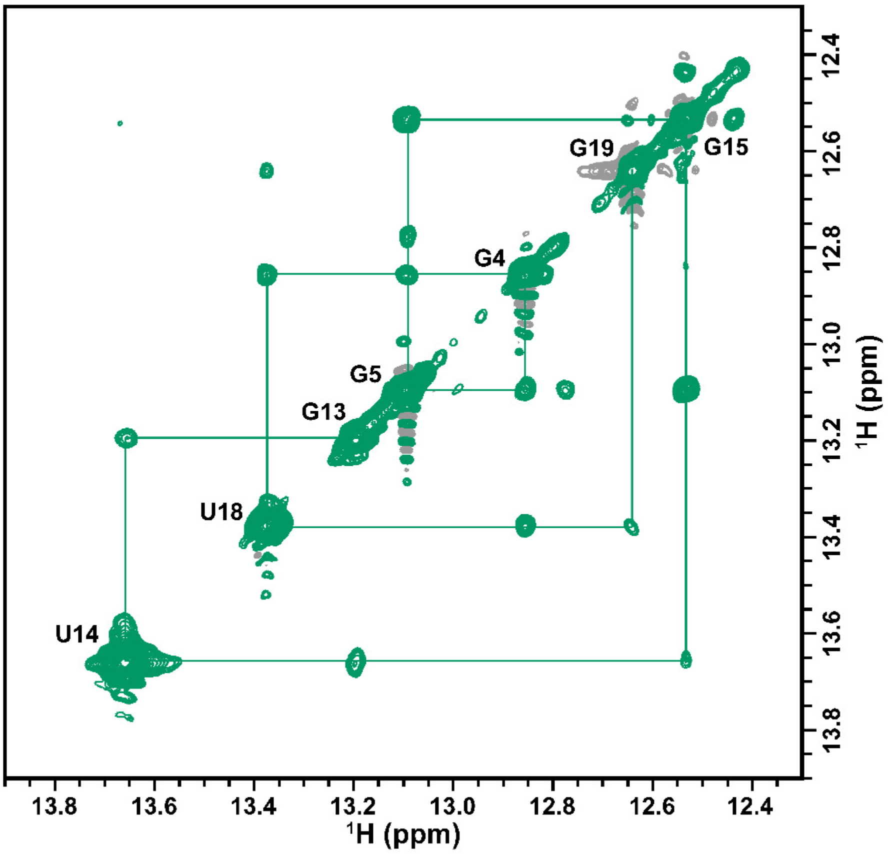

Supplementary Figure 1. Assignment of imino proton resonances of hpRNA20. NOESY spectrum recorded at 18°C with a 300 ms mixing time in 20 mM K-phosphate buffer (pH 6.5) containing 5% D_2O and 10 µM DSS. The green solid lines indicate imino proton–imino proton NOE connectivities.

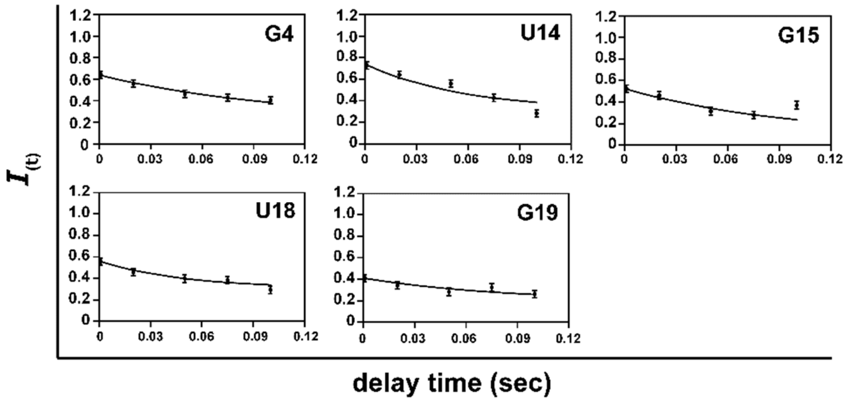

Supplementary Figure 2. Water magnetization transfer experiments on hairpin RNA (hpRNA20) in living HeLa cells. The relative peak intensities of imino protons, $I_{(t)}$, versus delay time for hpRNA20 in living HeLa cells. The intensity of imino proton was obtained for each residue (n = 1). Standard deviation of the noise signal for the region of 1D ^{1}H spectrum in which no signal is present was calculated and used as error bars of intensities. Source data are provided in the Source Data file.

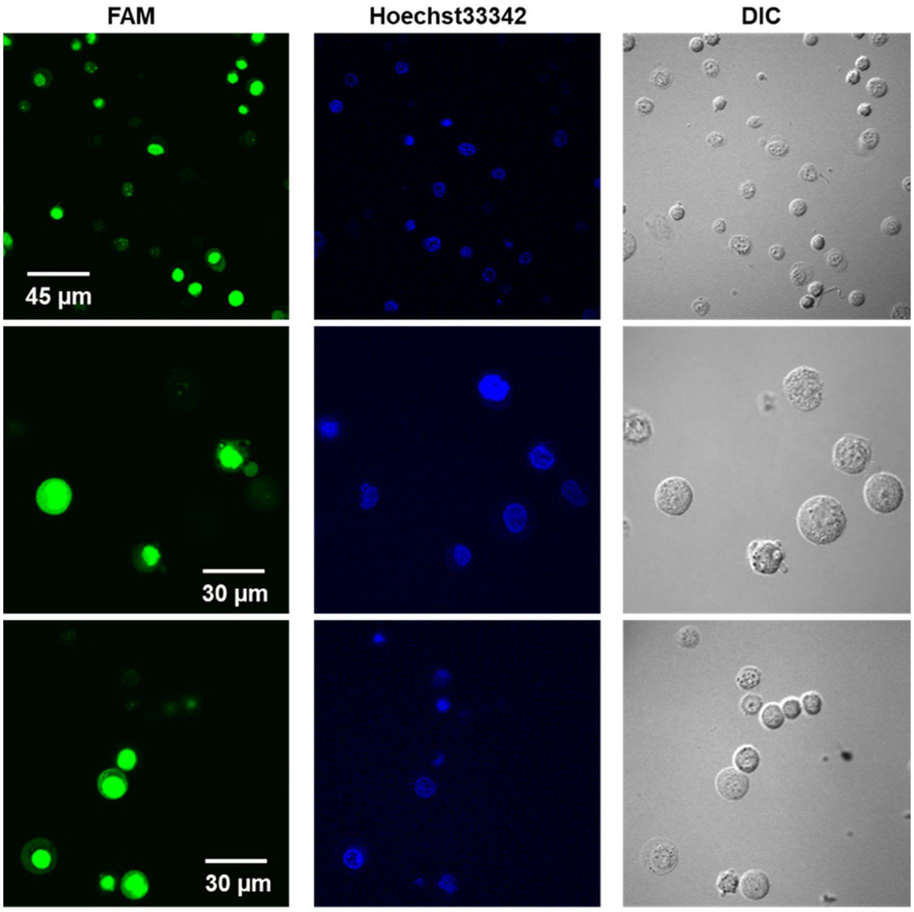

Supplementary Figure 3. Confocal fluorescence microscopy images and transmission images of FAM-teloDNA-introduced HeLa cells. (left) FAM-labeled teloDNA is detected. (middle) The nuclei are stained by Hoechst 33342. (right) A transmission image. n= 39 cells examined over 1 independent experiment.

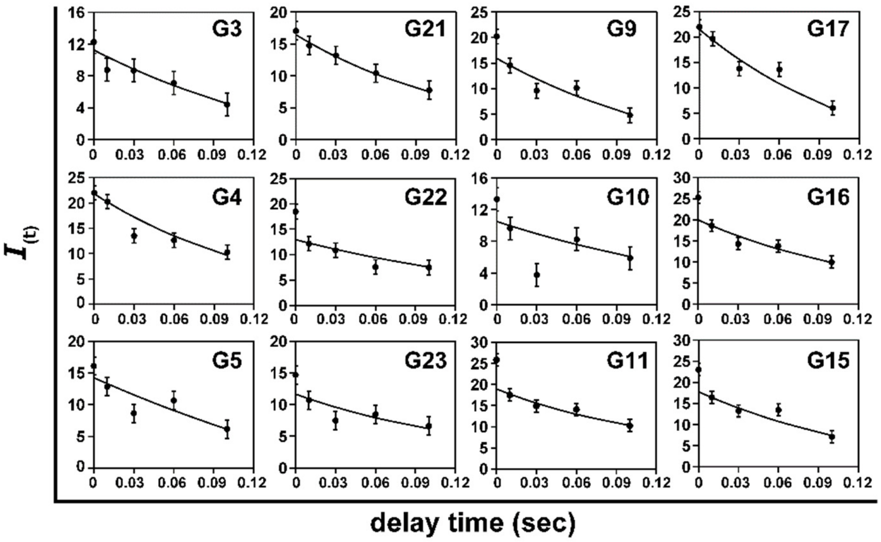

Supplementary Figure 4. Water magnetization transfer experiments on G-quadruplex DNA (teloDNA) in living HeLa cells. The relative peak intensities of imino protons, $I_{(t)}$, versus delay time for teloDNA in living HeLa cells. The intensity of imino proton was obtained for each residue (n = 1). Standard deviation of the noise signal for the region of 1D ^{1}H spectrum in which no signal is present was calculated and used as error bars of intensities. Source data are provided in the Source Data file.

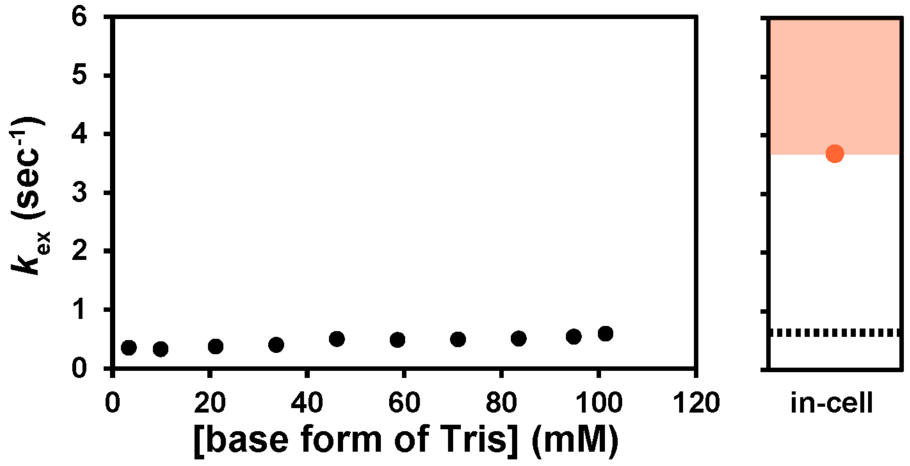

Supplementary Figure 5. The "average k_{ex} values" for teloDNA under *in vitro* and in-cell conditions. The dashed line indicates the maximum k_{ex} value for various concentrations of the base form of Tris. The maximum k_{ex} value equals $k_{\mathrm{open}}^{\mathrm{in\ vitro}}$. The orange shading indicates the deduced $k_{\mathrm{open}}^{\mathrm{in-cell}}$ value range for teloDNA in HeLa cells. Source data are provided in the Source Data file.

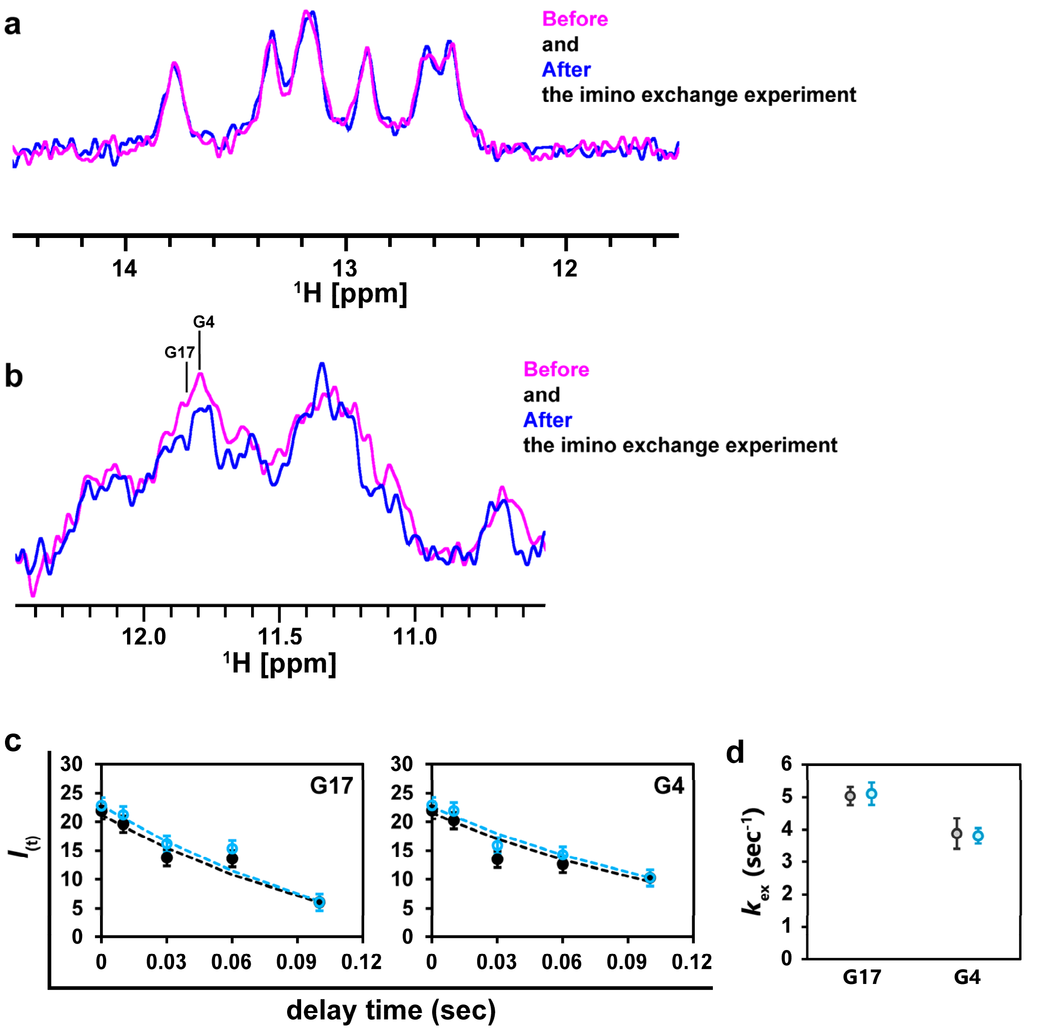

Supplementary Figure 6. Spectra before and after the in-cell water magnetization transfer experiment, and validation of the obtained $k_{\rm ex}$ values. a, b Superposition of the 1D ^{1}H spectra obtained before and after the imino exchange experiments for hpRNA20 (a) and teloDNA (b). **c** Curve fitting with (cyan) and without (grey) correction of the signal intensities for the determination of the $k_{\rm ex}$ values. **d** The $k_{\rm ex}^{\rm in-cell}$ values determined with (cyan) and without (grey) correction of the signal intensities. The intensity of imino proton was obtained for G17 and G4 (n = 1). Standard deviation of the noise signal for the region of 1D ^{1}H spectrum in which no signal is present was calculated and used as error bars of intensities (c). The means and error bars of the $k_{\rm ex}$ values were obtained from 50 data sets of intensities constructed by Monte Carlo simulation using the error bars of intensities. Data are presented as mean values ± standard deviation (d). Source data are provided in the Source Data file.

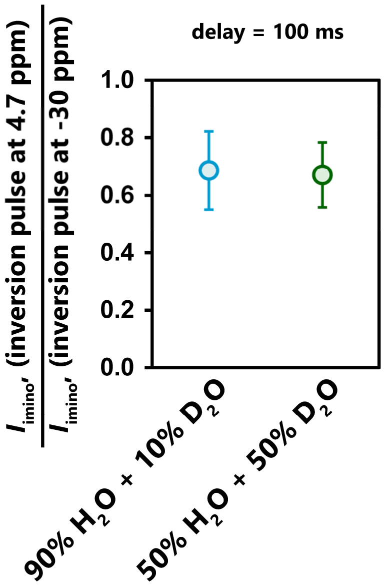

Supplementary Figure 7. The relative intensity of the imino-proton signals to inspect the effect of exchange-relayed NOEs from rapidly exchanging protons (mechanism (ii)). The imino-proton signals were obtained for the samples dissolved in either D_2O/H_2O=10%/90% or D_2O/H_2O=50%/50%. The intensity of imino proton was obtained under individual conditions (n = 1). The error bar of the relative intensity indicates the propagation error calculated from the error bars of each signal intensity that is the standard deviation of the noise signal for the region of 1D ^{1}H spectrum in which no signal. Source data are provided in the Source Data file.

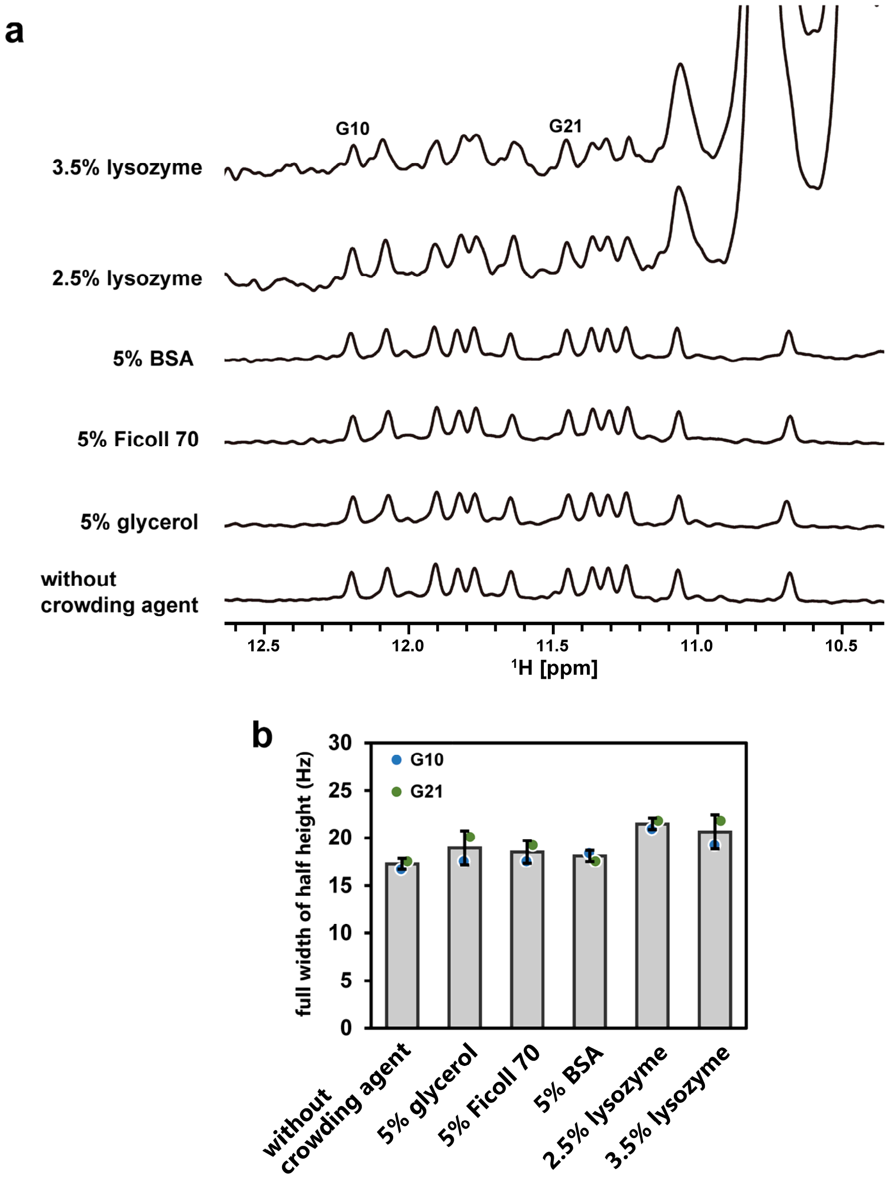

Supplementary Figure 8. The NMR spectra of teloDNA in the presence of crowding agents. **a** 1D ^{1}H-NMR spectra of teloDNA recorded in the presence of crowding agents. **b** The average of the full width of half height for the signals of G10 and G21 of teloDNA (n = 1). The error bars are the standard deviation. Source data are provided in the Source Data file.
